# Downregulation of USP9X in the DG Region of the Hippocampus Leads to AD‐Like Cognitive Dysfunction in Mice

**DOI:** 10.1111/cns.70493

**Published:** 2025-06-30

**Authors:** Xiaochuan Qi, Mengjiao Ying, Ao Wang, Kelin Shi, Guangshang Zhong, Yichao Lu, Changqing Liu, Yu Guo

**Affiliations:** ^1^ Anhui Engineering Research Centre for Neural Regeneration Technology and Medical New Materials Bengbu Medical University Bengbu China; ^2^ School of Laboratory Medicine Bengbu Medical University Bengbu China; ^3^ School of Life Sciences Bengbu Medical University Bengbu China; ^4^ Affiliated Hospital of Shandong University of Traditional Chinese Medicine, Pathology Jinan China; ^5^ School of Clinical Medicine Bengbu Medical University Bengbu China

**Keywords:** Alzheimer's disease, cognitive impairment, function research, neuronal dysfunction, USP9X

## Abstract

**Objective:**

This study investigates the link between ubiquitin‐specific peptidase 9 X‐linked (USP9X) and Alzheimer's disease (AD) pathogenesis, aiming to identify potential targets for AD diagnosis, treatment, and drug development.

**Methods:**

We constructed a USP9X‐inhibited expression mouse model and assessed cognitive and memory functions. We also measured Tau phosphorylation and APP levels in the hippocampal dentate gyrus (DG) and analyzed neuronal functions, dendritic spine features, late apoptosis, and autophagy.

**Results:**

Mice with USP9Xinhibition exhibited impaired cognitive and memory functions. An increase in the APP levels and Tau hyperphosphorylation in the DG resembled AD‐like pathology. Neurons exhibited abnormal functions, altered dendritic spine morphology, increase in neuronal apoptosis, and dysfunctions, similar to neuron loss observed in AD.

**Conclusion:**

Investigating the role of USP9X in AD could provide valuable insights for developing novel diagnostic and therapeutic strategies for AD.

## Graphical abstract

Experimental procedures at the animal and cell levels in this study.

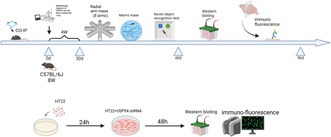

## 1 Introduction

Alzheimer's disease (AD), is the most prevalent neurodegenerative disease worldwide, primarily characterized by impaired cognitive decline, memory impairment, and progressive neuronal loss. Currently, AD affects approximately 55 million people globally; and with the rapid aging of populations, this number is projected to rise significantly, reaching 139 million by 2050 [1]. The pathological hallmarks of AD include three major structural alterations. First, the accumulation of β‐amyloid plaques between neurons disrupts synaptic communication [2]. Second, the hyperphosphorylation of tau protein leads to the formation of intracellular neurofibrillary tangles, impairing neuronal transport [3]. Finally, widespread neuronal death and synaptic loss lead to the collapse of the neuronal communication network, contributing to cognitive dysfunction [4].

Despite the widespread prevalence of AD and decades of research, currently no clear cure or treatment method exists. Present treatment is restricted to the use of prescription drugs for intervention; however, adverse reactions of the drugs such as, nausea, headache, and bleeding, result in limited efficacy. Moreover, the drugs merely alleviate the AD‐related symptoms, and do not provide any fundamental solution [5]. Therefore, exploring the pathogenesis of AD is an urgent need.

Ubiquitin‐specific proteases are the largest family of deubiquitinating enzymes, which regulate the ubiquitin‐proteasome system by specifically hydrolysing ubiquitin molecules in ubiquitinated proteins, thus, achieving a relative balance between ubiquitination and deubiquitination ensuring the proper regulation of protein homeostasis. This is crucial for preventing the accumulation of intracellular proteins [6]; especially in the central nervous system, it prevents neurological diseases by degrading and removing mutated and misfolded proteins (such as Tau, APP, and α‐synuclein) [7].

Ubiquitin‐specific protease USP9X (Recombinant Ubiquitin Specific Peptidase 9X, referred to as USP9X for short) is a highly conserved member of the USP family, located on chromosome Xp11.4 [8]. The catalytic domain contains a zinc finger motif whereas the finger subdomain contains three ubiquitin binding sites and a β‐hairpin insertion which contributes to the processing of polyubiquitin chains; the finger subdomain also contains the cleavage of Lysine (Lys) 11‐, Lys 63‐, Lys 48‐, and Lys 6‐bonds, enabling proteins to perform various cellular functions and exhibit multiple functions in different cellular processes [9, 10]. USP9X participates in multiple signal transduction pathways, including transforming growth factor‐β (TGF‐β), Hippo, Wnt/β‐catenin, and Janus kinase (JAK)‐STAT signal transduction pathways [11, 12, 13]. Recent studies have shown that abnormal expression of USP9X is closely related to a variety of neurodegenerative diseases and neurodevelopmental disorders [14]. Among the genes that were significantly altered in the public post‐mortem brain tissues of Alzheimer's disease (AD) patients, *USP9X* was included, and it showed a very significant gender expression difference in ad [ 15]. The deletion of USP9X in neurons can affect axonal growth and neuronal migration. Mice lacking USP9X have abnormal development of the subgranular zone (SGZ) of the hippocampal dentate gyrus after birth [16].

USP9X plays an important role in the imbalance of protein homeostasis caused by axonal transport defects in glaucoma and in the retrograde stress signal of axonal injury [7]. However, the molecular mechanism by which the alteration of USP9X expression leads to AD‐like cognitive and memory dysfunctions remains unclear. Therefore, in this study, USP9X‐suppressed expression adeno‐associated virus was used to reduce the expression of USP9X in the mouse hippocampus to explore the molecular mechanism of AD‐like changes and neuronal damage caused by USP9X‐suppressed expression, aiming to provide a new early diagnostic physiological target for AD.

## 2 Materials and Methods

### 2.1 Experimental Animals

Healthy female and male mice of the C57BL/6J strain were supplied by Hangzhou Ziyuan Laboratory Animal Technology Co. Ltd. [license number: SCXK(Zhe)2019‐0004]. The animals were housed in a clean‐grade animal room, with the environmental temperature controlled at 23°C ± 1°C, the relative humidity at 55%–65%, and the light–dark cycle at 12 h each. Experiments were carried out according to the requirements of the “Ethical Review Guidelines for Laboratory Animal Welfare in China (GB/T 35892‐2018),” and the welfare and ethics of laboratory animals were implemented in a standardized manner. The design and reporting of all experiments were in line with the “Animal Research: Reporting of In Vivo Experiments (ARRIVE)” guidelines. The use of experimental animals and all experimental procedures were approved by the Institutional Animal Care and Use Committee (IACUC) of Bengbu Medical College (approval number: 2024‐565).

### 2.2 Main Reagents

DMEM basic (Gibco), Paraformaldehyde (GENERAL‐REAGENT), OCT (SAKURA), Donkey Serum (Jackson Immuno Research), BSA (Solarbio), Triton‐100 (Sigma), DAPI (Beyotime), Solid Mounting Medium (Invitrogen), RIPA Lysis Buffer (Beyotime), Protease Inhibitor PMSF (Beyotime), Phosphatase Inhibitor (Bioss), PVDF Membrane (Millipore), Chemiluminescence Liquid (Millipore), Isoflurane (RWD), Immunohistochemistry Pen (BOSTER), Coverslip (Electron Microscopy Sciences), 0.25% Trypsin–EDTA (1×), Phenol Red (Gibco), Antibiotic‐Antimycotic (Gibco), PAGE Gel Rapid Preparation Kit (Shanghai Yasen Biotechnology), Fetal Bovine Serum (Corning), AAV‐USP9X‐shRNA (GenePharma), and LV3‐USP9X (GenePharma).

### 2.3 Co‐Immunoprecipitation (Co‐IP) and Western Blot Analyses

The brain tissue proteins of wild‐type C57 mice were lysed in IP buffer with protease inhibitor cocktail (Beyotime). Extracts were pre‐cleared with 20 μL protein A/G agarose beads for 1 h [17]. The protein lysates were incubated with RFWD2 or USP9X antibody on a rotator overnight at 4°C. Then, protein A/G agarose beads were added to combine the antigen/antibody complex for 2 min at room temperature(24°C–26°C). The magnetic beads were washed two times with immunoprecipitation lysis/wash buffer, and then once with pure water. Elute the antigen/antibody complex, and then perform Western Blot detection.

### 2.4 Stereotactic Injection of the Brain

Male C57BL/6J mice at 8 weeks of age and weighing 22–24 g were anesthetized with isoflurane gas. The experimental animals were divided into three groups: the blank control group (NC group, *n* = 30); the vehicle group injected with an empty virus at a speed of 0.1 μL/min into the bilateral hippocampal DG region (M/L: ±1.21 mm, A/P: −1.81 mm, D/V: −2.01 mm) using a brain stereotactic instrument according to the mouse brain atlas (*n* = 30); and the USP9X‐shRNA group injected with AAV9‐hSYN‐USP9X‐shRNA‐EGFP into the bilateral hippocampal DG region at the same speed (*n* = 30). Four weeks after injection, 6 mice from each group were randomly selected for administering euthanasia to extract brain tissue proteins and perform immunofluorescence staining. The remaining experimental animals in each group were subjected to behavioral tests by personnel unaware of the experiment.

### 2.5 Behavioral Tests of Mice

The mice in the blank control group (WT group, *n* = 16), the empty virus group (vehicle group, *n* = 16), and the USP9X suppressed expression group (USP9X‐shRNA group, *n* = 16) were placed in the breeding cages for adaptation for 5–7 days. Each behavioral test was conducted at intervals of one week and in the same quiet environment. After each animal completed the test, 75% alcohol was used to clean the equipment to remove the remaining odor information.

#### 2.5.1 Pole Test

Pole‐climbing equipment was put in a padded test box and the pole was fixed. Mice in each group were placed atop the pole, nose‐tip facing the pole's opposite side, to turn and climb down. The mice were adapted to the setup for 2 consecutive days. Formal tests were done on Day 3 and 4 to avoid mouse errors. Turning Time was the time recorded for the head‐nose tip to turn towards the climbing pole direction, and Down Time was the time recorded for the mouse to reach the pole bottom (*n* = 20 per group).

#### 2.5.2 Fatigue Rotarod Test

The rotarod was set at 20 revs/min for 10 min. Mice in each group were trained for 2 days to adapt to the setup. Formal tests were done on Day 3 and 4. After each mouse was tested, the max speed was set at 40 revs/min (uniform acceleration from 300 s to 40 revs) for 10 min, and the time mice stayed on the rod was recorded. For the on‐rod time test, it was set at 20 revs/min (uniform acceleration from 20 s to 20 revs) for 20 min, and the on‐rod time was recorded (*n* = 16 per group).

#### 2.5.3 Open Field Test

The size of the open field test box for mice is 50 × 50 × 40 cm. Prior to 24 h of the test or training, mice of each group were placed in the test room to adapt to the test environment. The animals were gently placed in the centre of the test box to allow free exploration for 10 min. For each experiment, the animals were then placed in the centre of the test box at the same position and in the same direction (*n* = 20 for each group) [18].

#### 2.5.4 Novel Object Recognition Test

This test has two stages. In the first old‐object exploration stage, objects A and B (same material and size) were placed 12.5 cm from the wall in an open‐field box. Mice, placed with backs to the objects, moved freely for 10 min. Exploration (nose/mouth touches, time within 2 cm) was video‐recorded. After a 1‐h break, the second stage started. Object B was replaced with object C of a different shape. The operation was as in the first stage (*n* = 12 per group). The ratios of new‐to‐old object exploration times and frequencies were used as recognition indices (TRI and NRI) [19].

#### 2.5.5 Morris Water Maze Assay (MWM) Test

The facility comprises a circular pool with a bottom heating rod that maintains the water temperature at about 26°C. The chamber is surrounded by curtains, has spatial markers, and an escape platform. In the adaptation stage on Day 1, the water was kept clear, and the platform was raised 1 cm above water for mice to find and remember. During the following 6‐day training stage, the platform was kept underwater, and titanium dioxide was used to make the water turbid. Mice were placed in water from four directions daily, with a 60‐s exploration limit. If the mice failed to find the platform, a plastic stick guided them to it for a 15‐s stay. From Day 8, formal tests were done two times in a day. Mice were placed in water at the point farthest from the platform. A camera recorded the trajectory of the mice and the time each mouse required to find the platform. Each mouse was tested three times, with at least 2 min intervals between the experiments; the durations of the three repeated experiments were added up for statistical plotting (*n* = 20 per group) [20].

#### 2.5.6 Eight‐Arm Maze Experiment

The mice were first adapted to the experimental setup for 3 days; they were not provided with food for 24 h, and then restricted quantities of food were provided daily. From Days 4 to 10 (training phase), food was randomly put in 4 arms. Mice were placed at the centre to forage for 10 min. On Day 7, open the electric heating pad device in the punishment arm. Set the temperature of the electric heating punishment device to 60°C. Only place the electric heating device in the electric heating punishment arm and not the food (the application of foot heat stimulation at 60°C had no significant effect on the motor ability of mice, proving that compared with other strong stimuli such as water, light, and electricity, heat stimulation at a reasonable temperature could better reduce the stress probability of the test animals to avoid experimental failure [21]), and training was repeated two times with a 1‐h interval. During Days 11–15 (testing phase), food was placed in the chamber and mice were placed at the centre for 30 s, then the door was opened for feeding. Tests ended if food was not eaten within 10 min from the start of the experiment. Arm‐entry numbers were recorded daily. A 5‐day memory curve, from Day 11 onward, was plotted (*n* = 16/group). Visiting an arm with previously food was a working error; an arm without food was a reference memory error. After tests, ANY‐Maze 7.1.6 analyzed videos and statistics [22].

### 2.6 Immunofluorescence Staining of Brain Tissue

The mice in the WT group, vehicle group, and USP9X‐shRNA group were perfused with pre‐cooled PBS, followed by 4% paraformaldehyde under deep anesthesia by isoflurane gas. The mouse brains were taken out and fixed overnight in 4% paraformaldehyde, then gradient dehydration was performed using 15% sucrose to 30% sucrose, and frozen at −80°C overnight. Perfused brains were sectioned into 12‐μm thick coronal sections using a microtome cryostat (Leica, CM‐1850, Wetzlar, Germany) and mounted on gelatine‐coated glass slides. The cryosections were blocked with a mixture of PBS, donkey serum, 2% BSA, and 10% Triton‐100 for 1 h, rinsed with PBS, and then incubated overnight at 4°C with rabbit anti‐USP9X (Abcam 1:500), mouse anti‐USP9X (Santa Cruz 1:500), mouse anti‐c‐Fos (Santa Cruz 1; 200), mouse anti‐P62 (Santa Cruz 1; 500), mouse anti‐CD68 (Invitrogen 1:500), rabbit anti‐Iba1 (Cell Signaling Technology 1:500), rabbit anti‐PSD95 (Cell Signaling Technology 1:500), and mouse anti‐Bcl‐2 (Santa Cruz 1:500). The corresponding secondary antibodies Alexa Fluor 488 (1:500), Alexa Fluor Cy3 (1:1000), and Alexa Fluor 647 (1:1000) were added and incubated for 1 h. The nuclei were counterstained with 4′,6‐diamidino‐2‐phenylindole dihydrochloride (DAPI) for 15 min. The immunofluorescence results were observed under a multiphoton laser scanning microscope (Olympus, FV1200MPE SHARE, Tokyo, Japan).

Golgi staining: Fresh brain tissue was fixed in 4% paraformaldehyde. Hippocampal blocks were cut per brain atlas. After rinsing, they were treated with a Golgi staining solution for 14 days (with solution changes). They were then immersed in distilled water, softened with glacial acetic acid, treated with sucrose solution, sliced by vibrating microtome, and attached to a gelatine‐treated slide to dry. Next, they were treated with gelatine‐potassium chromic sulphate mix and concentrated ammonia before drying and mounting with glycerol gelatine. Finally, a digital slice scanner was used to capture the brain tissue panoramic image. Ten neurons in the hippocampal DG region (same area) from WT and USP9X—shRNA groups were randomly selected for dendritic spine density and morphology analysis.

Nissl staining: Fresh brain tissue was fixed in 4% paraformaldehyde, dehydrated using alcohol of gradient concentrations, and then made transparent using xylene. After embedding in paraffin and sectioning, Nissl staining was performed. Post dewaxing, the samples were observed under a microscope. The WT group and the USP9X‐shRNA group underwent analysis of the gray value of the depth of Nissl staining and statistics of the number of cells.

### 2.7 Cell Culture and Construction of Lentivirus Transfected HT‐22 Cell Line

The lentivirus for suppressing the expression of USP9X was constructed by GenePharmar Company, with Puro resistance of LV3‐USP9X, and its sequence from 5′ to 3′: GCAGAAGAAATCACTATGATT. Mouse hippocampal neuron HT22 cells (provided by Cell Bank of the Centre for Excellence in Molecular Cell Science, Chinese Academy of Sciences) were cultured and seeded in 96‐well plates of about 0.5–1 × 10^4^ cells/well. The next day, the previous cell culture medium was removed, and the new medium containing 5 μg/mL Polybrene and gradient‐diluted virus was added. After 48 h of infection, puromycin was added to remove the cells that were not successfully infected. After 24 or 48 h, the fresh complete medium was replaced for continuous culture. The optimal MOI value of virus infection was detected by fluorescence microscopy. According to the optimal MOI value, the above operations were repeated, and the expanded culture of infected cells was carried out.

### 2.8 Western Blot Detection

Total proteins were extracted from the hippocampal tissues of mice in the WT group, vehicle group, and USP9X‐shRNA group using the total protein extraction kit (Beyotime) and quantified using a BCA protein assay kit (Beyotime). Equivalent quantities of protein (40 μg/sample) were subjected to SDS‐PAGE electrophoresis and transferred to the PVDF membranes (0.45 μM; MilliporeSigma). Then, the membranes were incubated overnight at 4°C with the primary antibodies of rabbit anti‐USP9X (Abcam 1:5000), rabbit anti‐PSD95 (Cell Signaling Technology 1:5000), rabbit anti‐SYN (Abcam 1:5000), rabbit anti‐Vglut1 (Invitrogen 1:3000), mouse anti‐GAD67 (Santa Cruz 1:1000), mouse anti‐P62 (Santa Cruz 1:1000), rabbit anti‐LC3B (BIOS 1:1000), mouse anti‐Beclin1 (Santa Cruz 1:3000), mouse anti‐Bcl‐2 (Santa Cruz 1:1000), rabbit anti‐BAX (protein tech 1:8000), rabbit anti‐Caspase3 (protein tech 1:2000), and rabbit anti‐Caspase9 (protein tech 1:1000). The next day, the samples were incubated with IgG‐HRP antibody (1:5000) at room temperature (24°C–26°C) for 1 h. The protein bands were then detected by Clarity Western ECL Blotting Substrates (Bio‐Rad Laboratories, Hercules, CA, USA) and quantified using Software Quantity One version 1‐D (Bio‐Rad Laboratories).

### 2.9 Cell Immunofluorescence Staining

The cells cultured on the coverslips were fixed with 4% PFA for 18 min, permeabilized with 0.2% Triton X‐100 for 10 min, and blocked with 1% bovine serum albumin (BSA) and 10% normal donkey serum (NDS) at room temperature (24°C–26°C) for 1 h. The samples were incubated with the following primary antibodies at 4°C overnight using rabbit anti‐USP9X (Abcam 1:500), mouse anti‐USP9X (Santa Cruz 1:500), rabbit anti‐PSD95 (Cell Signaling Technology 1:5000), rabbit anti‐Vglut1 (Invitrogen 1:200), mouse anti‐GAD67 (Santa Cruz 1:300), mouse anti‐P62 (Santa Cruz 1:500), rabbit anti‐LC3B (BIOS 1:500), mouse anti‐Bcl‐2 (Santa Cruz 1:500), and rabbit anti‐Caspase9 (protein tech 1:500) overnight. The samples were incubated with secondary antibodies Alexa Fluor 488 (1:500) and Cy3 (1:1000) for 1 h in the dark at room temperature(24°C–26°C), and the results were observed under a fluorescence microscope.

### 2.10 Statistical Analysis and ImageJ Image Analysis

All data were analyzed using SPSS 26.0 software and visualized using GraphPad Prism 8.0 software from at least three independent experiments. For all the data, we employed the Shapiro–Wilk test to assess their normality. Differences between the two groups were analyzed using the two‐independent‐sample *t*‐test. For the comparison of differences among multiple samples, one‐way ANOVA and LSD (least significant difference) was used for pairwise comparisons if they conformed to normal distribution and homogeneity of variance; for cases that did not conform to normal distribution, the non‐parametric test method was used, whereas for multiple independent samples, the Kruskal–Wallis test was used. A difference was considered statistically significant at *p* < 0.05. The analysis of dendritic spine density, morphology, and the gray value of Nissl staining was performed using the ImageJ software.

## 3 Results

### 3.1 Inhibition of USP9X Expression in the DG Region of the Hippocampus Causes Cognitive and Memory Dysfunctions in Mice

**FIGURE 1:**
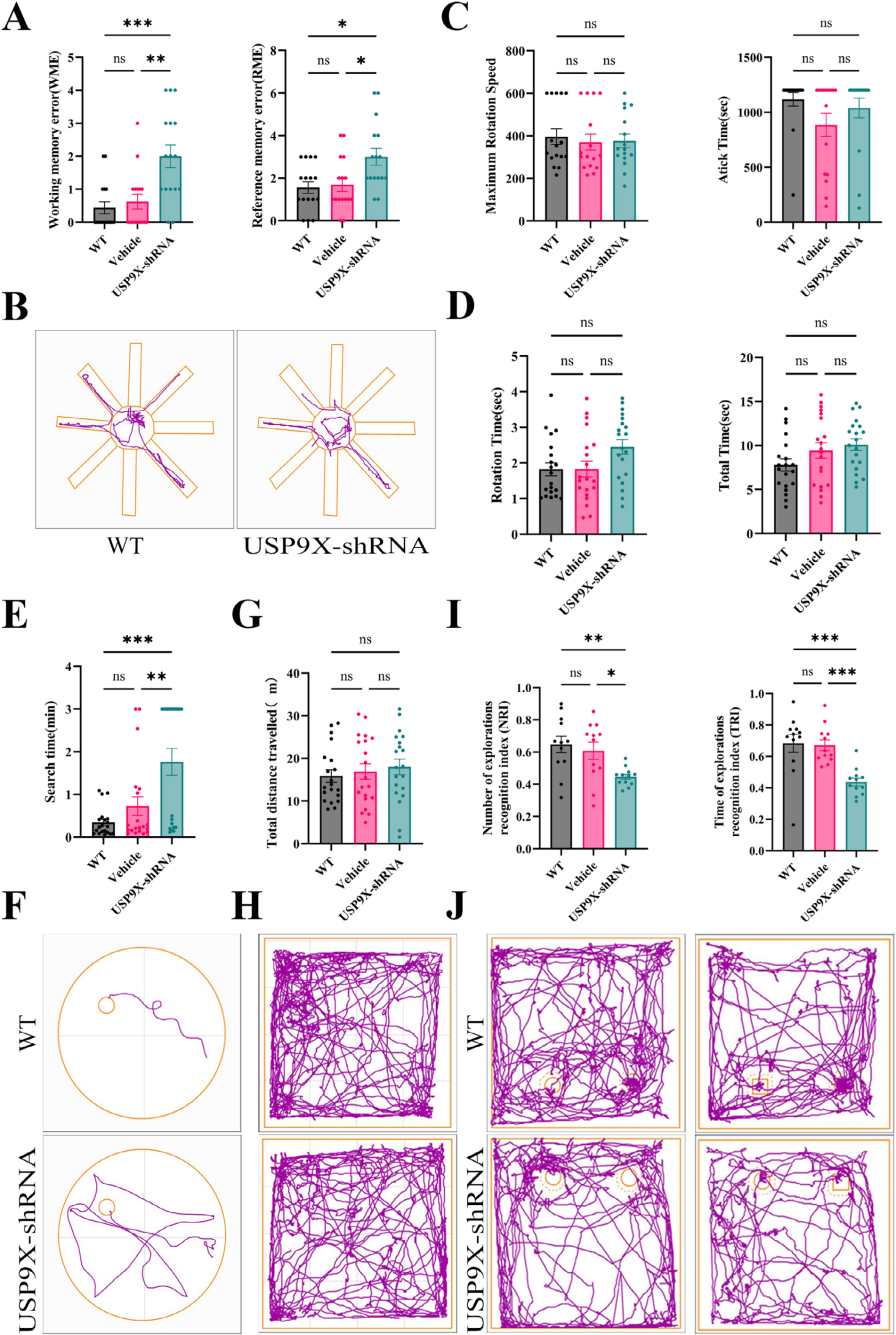
Inhibition of USP9X expression in the hippocampal DG region leads to cognitive and memory dysfunction in mice. (A, B): evaluation of working memory error (WME) and reference memory error (RME) indicators, and movement trajectory maps of mice in the testing stage of the eight‐arm maze (*n* = 16 for each group); (C): evaluation of the maximum rotational speed and fixed rotational speed in the fatigue rotarod test (*n* = 16 for each group); (D): evaluation of pole test rotation time and total time (*n* = 20 for each group); (E, F): evaluation of the time to find the platform in the water maze and movement trajectory maps (search time *n* = 20); (G, H): total distance moved by mice in the open field test of mice; movement trajectory of mice (*n* = 20); (I, J): recognition index of the number of exploration times for new and old objects (NRI) and movement trajectory maps, recognition index of the exploration time for new and old objects (TRI), and movement trajectory maps (*n* = 12). *p < 0.05, **p < 0.01, ***p < 0.001.

AAV9‐hSYN‐USP9X‐shRNA was stereotactically injected into the DG region of the mouse hippocampus to inhibit the expression of USP9X. Four weeks later, behavioral tests were conducted to determine whether the cognitive, memory, and motor functions of mice with inhibited USP9X expression were impaired. The results of the Pole Test and the fatigue rotarod test showed that the time of maximum rotation, the time on the rod, rotation time, and total time of mice in the USP9X‐shRNA group did not decrease significantly compared to those in the WT and Vehicle groups (in Figure 1C, on the left side, *F* (2, 45) = 0.1481 and *p* = 0.8627, and on the right side, *F* (2, 45) = 1.822 and *p* = 0.1734, with no significant difference. In Figure 1D, on the left side, *F* (2, 57) = 3.135 and *p* = 0.0511, and on the right side, *F* (2, 57) = 2.499 and *p* = 0.0912, with no significant difference). Moreover, the results of the open field tests showed that the total distance traveled by mice with inhibited USP9X expression is not significantly different compared to that of the WT mice (in Figure 1G *F* (2, 57) = 0.3950, *p* = 0.6755, and there is no significant difference), indicating that the downregulation of USP9X in the DG region of the mouse hippocampus did not cause obvious motor dysfunction.

The number of WME and RME in USP9X‐shRNA mice significantly increased, indicating that the downregulation of USP9X in the DG region of the mouse hippocampus impaired both the long‐term memory (spatial memory ability) and short‐term memory (working memory) of mice (in the left side of Figure 1A *F* (2, 45) = 10.99 and *p* = 0.0001. In the right side, *F* (2, 45) = 5.752 and *p* = 0.0060, showing a significant difference). The results of the Morris water maze test indicated that, compared with WT mice, the mice with inhibited USP9X expression took a significantly longer time to find the platform, and their movement trajectories were also characterized by circling along the edges. Thus, inhibiting the expression of USP9X in the hippocampal DG region significantly impaired the spatial memory ability of mice (in Figure 1E *F* (2, 57) = 10.69 and *p* = 0.0001, and there is a significant difference). In the novel object recognition test, the number and time of exploration of the new object by mice in the USP9X inhibition expression group decreased (in Figure 1I, on the left side, *F* (2, 33) = 5.966 and *p* = 0.0061, and on the right side, *F* (2, 33) = 11.42 and *p* = 0.0002, with a significant difference), indicating that the cognitive ability of mice with inhibited USP9X expression was significantly impaired.

### 3.2 Inhibition of USP9X Expression Causes Pathological Changes Similar to AD in Mice

**FIGURE 2:**
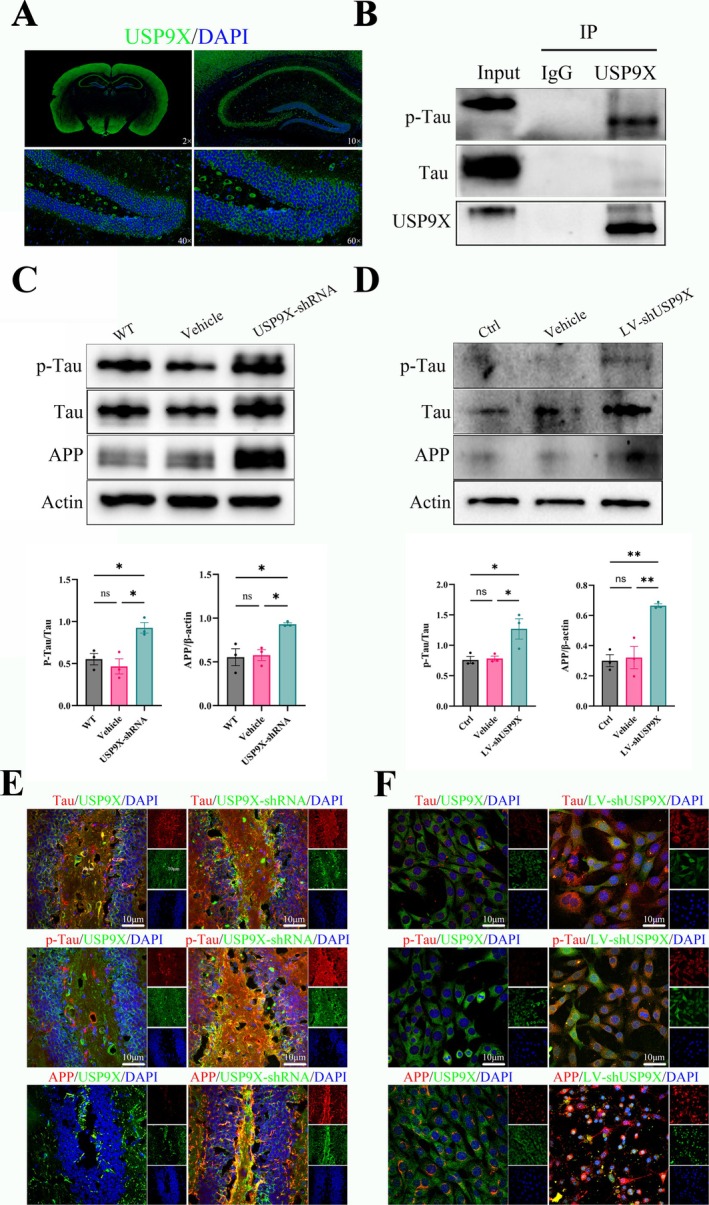
Decreased expression of USP9X causes abnormal deposition of a β amyloid protein. (A): localization of USP9X in the whole brain of mouse; (B): immunoprecipitation results of USP9X; (C): western blot results and quantification of hippocampal tissue with inhibited USP9X expression; (D): western blot results and quantification of downregulated USP9X expression in HT22 cells; (E): immunofluorescence staining related to Aβ amyloid protein in the hippocampal region; (F): cell immunofluorescence staining. *p < 0.05, **p < 0.01.

The results of co‐localization and co‐immunoprecipitation (CO‐IP) of USP9X in the whole brain showed that USP9X was expressed throughout the mouse brain (Figure 2A), and an interaction between USP9X and Tau and p‐Tau proteins existed (Figure 2B). After stereotactic brain injection of AAV9‐hSYN‐USP9X‐shRNA, compared with WT mice, the phosphorylation of Tau protein increased, and amyloid precursor protein also significantly increased (in Figure 2C, for p‐Tau/Tau, *F* (2, 6) = 10.74 and *p* = 0.0104; for APP/β‐actin, *F* (2, 6) = 9.936 and *p* = 0.0125, and there are significant differences). The co‐localization regions of Tau, p‐Tau, and APP as indicated by immunofluorescence also significantly increased (Figure 2E). The immunofluorescence results were consistent with the western blot results. At the cellular level, the expression of USP9X was reduced by infecting LV‐USP9X‐shRNA. The results of western blot and immunofluorescence were consistent with the conclusions drawn at the tissue level (in Figure 2D, for p‐Tau/Tau, *F* (2, 6) = 7.557 and *p* = 0.0229; for APP/β‐actin, *F* (2, 6) = 17.55 and *p* = 0.0031, and there are significant differences).

### 3.3 Downregulation of USP9X Causes Negative Changes in the Morphology and Quantity of Dendritic Spines in Mouse Hippocampal Neurons

**FIGURE 3:**
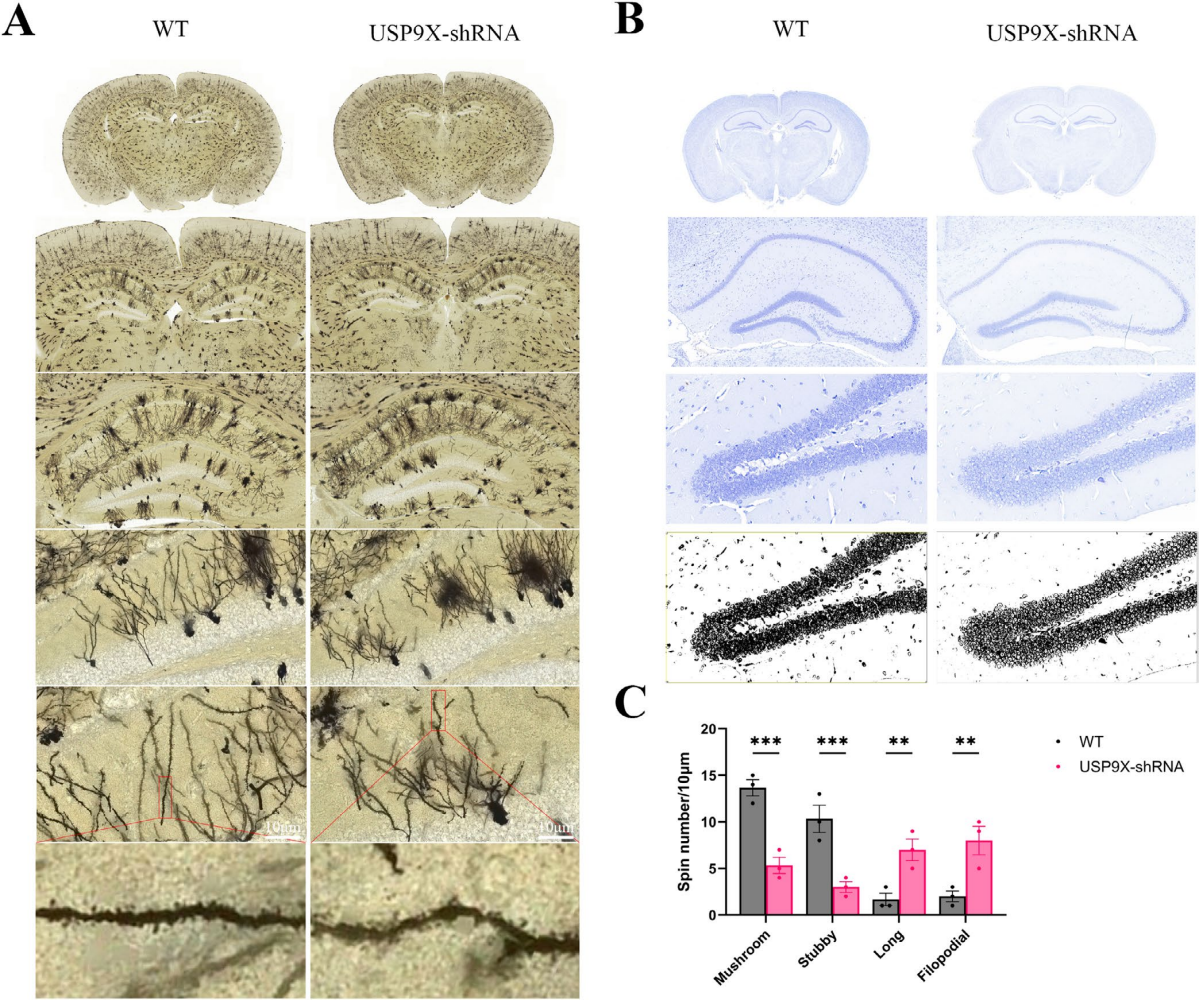
Golgi staining and Nissl staining of brain tissue after inhibiting USP9X expression. (A): representative images of whole brain scanning with Golgi staining after injecting AAV9‐hSyn‐USP9X‐shRNA into the DG region of mouse hippocampus for 4 weeks (120×); (B): representative images of whole brain scanning with Nissl staining after injecting AAV9‐hSyn‐USP9X‐shRNA into the DG region of mouse hippocampus for 4 weeks (600×); (C): statistics of the proportion of dendritic spine morphology in Golgi staining. **p < 0.01, ***p < 0.001.

Four weeks after injecting AAV9‐hSYN‐USP9X‐shRNA into the DG region of the mouse hippocampus, the Golgi staining results showed that post the downregulation of USP9X in the DG region of the hippocampus of C57 mice, the dendritic branches of hippocampal neurons decreased, and dendritic complexity decreased; this manifested as a significant reduction in mature dendritic spines (mushroom‐shaped and stubby‐shaped), and a significant increase in immature (slender‐shaped and filopodia‐shaped) dendritic spines. The morphology, quantity, and density of dendritic spines changed significantly (Figure 3A). Therefore, inhibition of USP9X expression has a significant impact on dendritic spine formation and function (in Figure 3C, for Mushroom, *T* = 5.735 and *p* = 0.0001; for Stubby, *T* = 5.047 and *p* = 0.0005; for Long, *T* = 3.671 and *p* = 0.0082; for Filopodial, *T* = 4.129 and *p* = 0.0031, and there are significant differences). The results of Nissl staining showed that in the WT group, the Nissl bodies were larger in volume, more in number, and darker in staining, and the physiological state of neurons was normal (Figure 3B). However, the number of Nissl bodies in the hippocampal neurons with downregulated USP9X decreased or disappeared, and the staining became lighter and blurred (Figure 3B), indicating that the function of nerve cells in the DG region of the mouse hippocampus to synthesize proteins decreased significantly after the downregulation of USP9X.

### 3.4 The Downregulation of USP9X in Hippocampal Neurons Did Not Cause the Proliferation of Glial Cells and the Occurrence of Neuroinflammation

**FIGURE 4:**
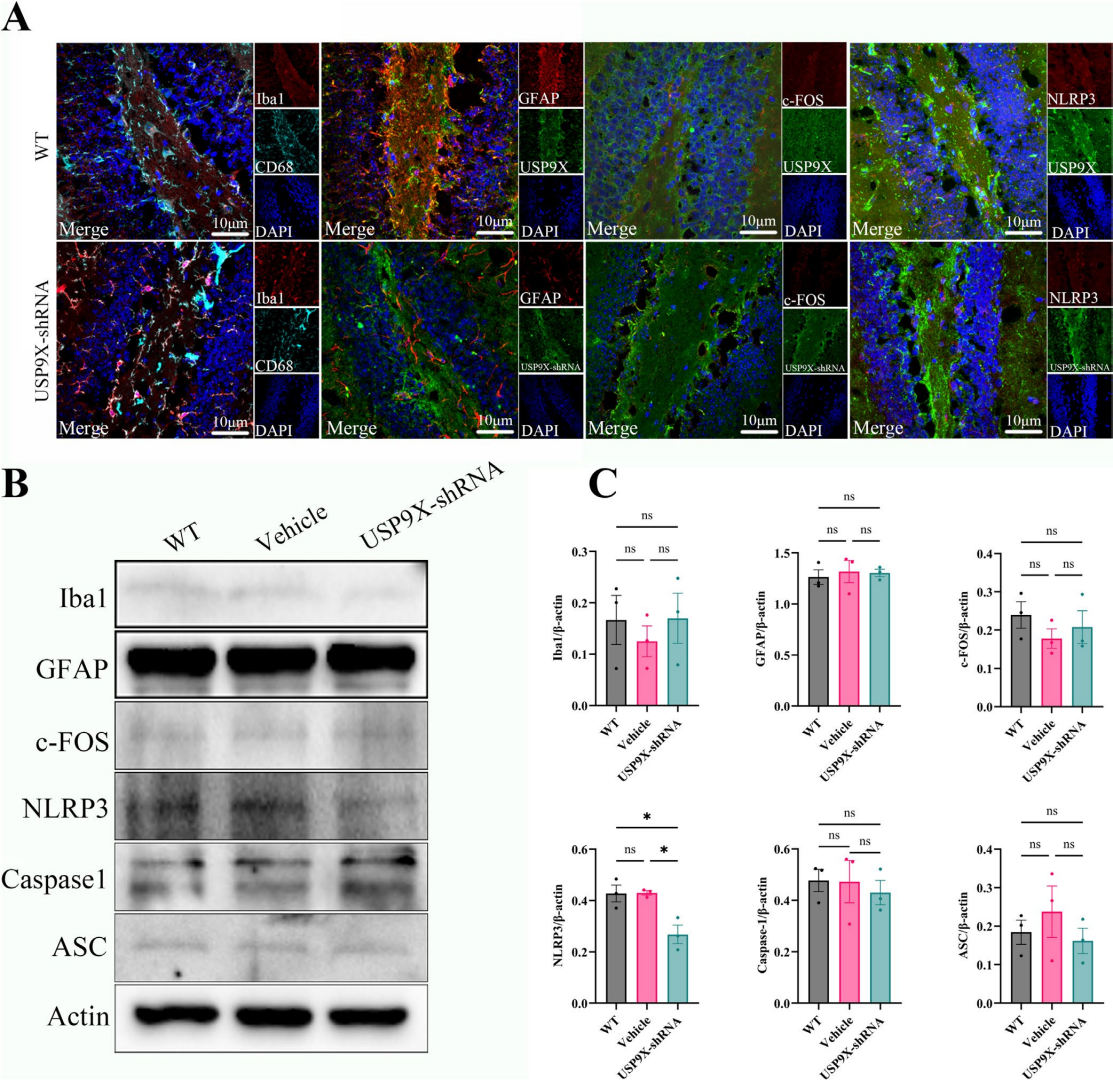
The effect of inhibiting USP9X expression on neuroinflammation in the DG region of the hippocampus. (A): immunofluorescence staining of microglial marker Iba1, activated microglial marker CD68, astrocyte marker GFAP, and neuronal activity biomarker c‐Fos; (B, C): western blot results and quantification of neuroinflammation‐related markers of mouse hippocampal tissue proteins. *p < 0.05.

To explore whether injecting AAV9‐hSYN‐USP9X‐shRNA into the DG region of the mouse hippocampus causes acute inflammation and glial cell proliferation in brain tissue, acute brain injury‐related immunofluorescence staining was performed. The results showed that compared with the WT group, the expression of the activated microglial marker CD68 and the microglial marker Iba1 did not increase significantly in the vehicle and USP9X‐shRNA group, and GFAP as the astrocyte marker did not increase significantly either, indicating that microglial cells did not show significant activation, and the number of astrocytes did not increase significantly (Figure 4A). The expression of the typical biomarker of neuronal activity, c‐Fos protein, did not show a significant difference, and the inflammasome NLRP3 did not show significant activation either (Figure 4A). Moreover, the results of western blot analysis were consistent with the immunofluorescence results (in Figure 4B,C, for Iba1/β‐actin, *F* (2, 6) = 0.3348 and *p* = 0.7280; for GFAP/β‐actin, *F* (2, 6) = 0.1297 and *p* = 0.8808; for c‐Fos/β‐actin, *F* (2, 6) = 0.7824 and *p* = 0.4989; for NLRP3/β‐actin, *F* (2, 6) = 10.57 and *p* = 0.0108; for Caspase1/β‐actin, *F* (2, 6) = 0.1841 and *p* = 0.8364; for ASC/β‐actin, *F* (2, 6) = 0.6915 and *p* = 0.5367, and there are no significant differences). Therefore, the downregulation of USP9X in the DG region of the mouse hippocampus did not cause acute brain injury and glial cell proliferation, excluding the possibility that changes in mouse behavioral paradigms were caused by brain injury inflammation.

### 3.5 Downregulation of USP9X in Hippocampal Neurons Causes Changes in Neuronal Synaptic Proteins

**FIGURE 5:**
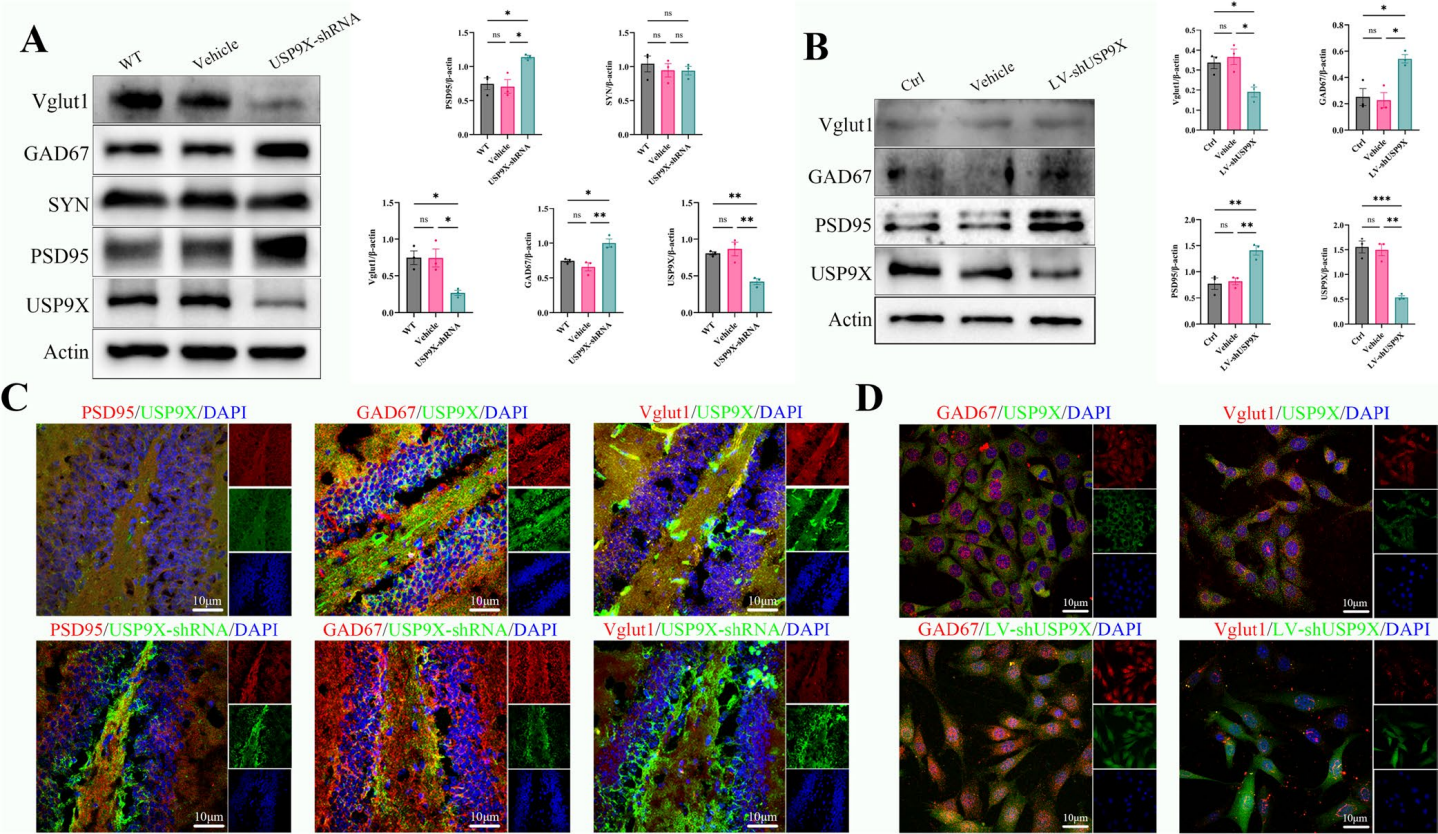
The effect of inhibiting USP9X expression on neurotransmitters of hippocampal neurons in the DG region. (A, B): western blotting results and quantitative analysis of mouse hippocampal tissue proteins and cell proteins; (C): immunofluorescence staining of brain tissue sections; (D): cell immunofluorescence staining (GAD67: marker of GABA‐ergic inhibitory interneurons; PSD95: postsynaptic membrane density protein; SYN: presynaptic membrane vesicle protein; Vglut1: marker of glutamatergic excitatory interneurons). *p < 0.05, **p < 0.01, ***p < 0.001.

After the inhibition of USP9X expression in the mouse hippocampal region, it was investigated whether the connection and excitability between neurons in the DG region were affected. The results showed that the downregulation of USP9X caused a significant increase in the postsynaptic membrane protein PSD95 and the inhibitory interneuron marker GAD67, while the excitatory neuron marker Vglut1 significantly decreased, indicating that the inhibitory expression of USP9X weakened the synaptic connection between hippocampal neurons and inhibited neuronal function (in Figure 5A, for Vglut1/β‐actin, *F* (2, 6) = 9.323 and *p* = 0.0144; for GAD67/β‐actin, *F* (2, 6) = 12.41 and *p* = 0.0074; for PSD95/β‐actin, *F* (2, 6) = 9.306 and *p* = 0.0145; for USP9X/β‐actin, *F* (2, 6) = 16.84 and *p* = 0.0035, and there are significant differences. For SYN/β‐actin, *F* (2, 6) = 0.3591 and *p* = 0.7124, and there is no significant difference). The immunofluorescence of brain tissue sections showed that the co‐localization of PSD95, GAD67, and USP9X‐shRNA significantly increased, and the decrease in the co‐localization of Vglut1 also confirmed this conclusion (Figure 5C). To reduce interference factors, verification was carried out at the HT22 cell level using western blotting and immunofluorescence, and the results were consistent with those at the animal level (in Figure 5B, for Vglut1/β‐actin, *F* (2, 6) = 8.539 and *p* = 0.0176; for GAD67/β‐actin, *F* (2, 6) = 10.90 and *p* = 0.0101; for PSD95/β‐actin, *F* (2, 6) = 15.97 and *p* = 0.0040; for USP9X/β‐actin, *F* (2, 6) = 33.76 and *p* = 0.0005, and there are significant differences).

### 3.6 Downregulation of USP9X Causes an Increase in Hippocampal Neuronal Apoptosis

**FIGURE 6:**
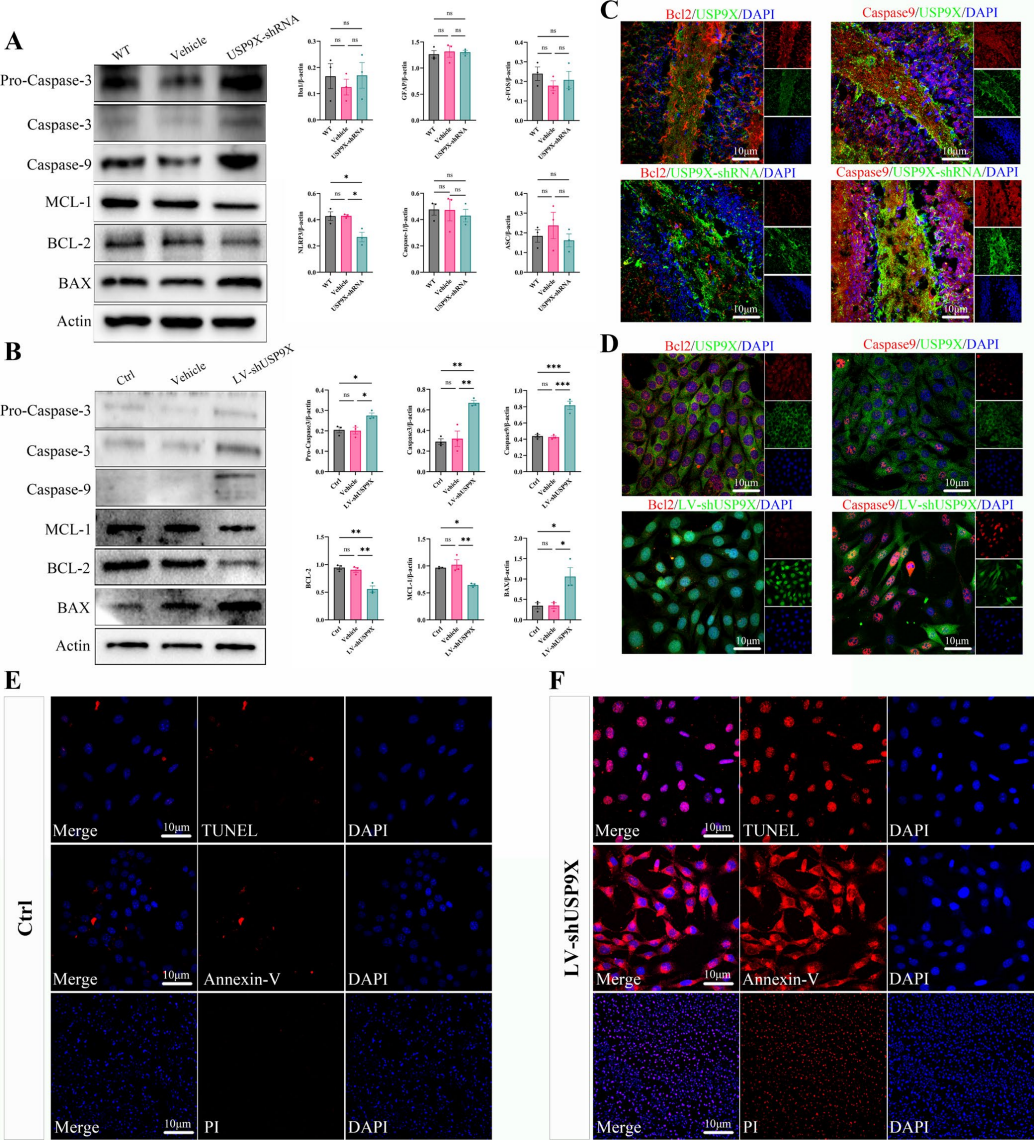
The effect of downregulation of USP9X expression on hippocampal neuronal apoptosis. (A): western blotting detection results and quantification of apoptosis‐related markers of tissue proteins; (B): apoptosis western blotting detection results and quantification at the HT22 cell level; (C): tissue immunofluorescence staining of apoptotic proteins; (D): cell immunofluorescence results; (E, F): results of apoptosis‐related immunofluorescence staining. *p < 0.05, **p < 0.01, ***p < 0.001.

The western blotting results of the mouse hippocampal tissue proteins with AAV9‐hSYN‐USP9X‐shRNA and HT‐22 cells infected with LV‐USP9X‐shRNA showed that after the reduction of USP9X expression, the expressions of pro‐apoptotic proteins Caspase‐3, Caspase‐9, and BAX were significantly upregulated, and the expressions of anti‐apoptotic proteins Bcl‐2 and MCL‐1 were significantly decreased (in Figure 6A, for Pro‐Caspase3/β‐actin, *F* (2, 6) = 7.813 and *p* = 0.0214; for Capase3/β‐actin, *F* (2, 6) = 28.92 and *p* = 0.0008; for Caspase9/β‐actin, *F* (2, 6) = 18.10 and *p* = 0.0029; for MCL‐1/β‐actin, *F* (2, 6) = 24.88 and *p* = 0.0012; for BCL‐2/β‐actin, *F* (2, 6) = 8.960 and *p* = 0.0158; for BAX/β‐actin, *F* (2, 6) = 11.11 and *p* = 0.0096, and there are significant differences. In Figure 6B, for Pro‐Caspase3/β‐actin, *F* (2, 6) = 8.290 and *p* = 0.0188; for Capase3/β‐actin, *F* (2, 6) = 19.24 and *p* = 0.0025; for Caspase9/β‐actin, *F* (2, 6) = 45.35 and *p* = 0.0002; for MCL‐1/β‐actin, *F* (2, 6) = 13.71 and *p* = 0.0058; for BCL‐2/β‐actin, *F* (2, 6) = 21.87 and *p* = 0.0018; for BAX/β‐actin, *F* (2, 6) = 8.965 and *p* = 0.0158, and there are significant differences). The tissue immunohistochemistry and cell immunofluorescence staining showed that after the inhibition of USP9X expression, the co‐localization area of Bcl‐2 and USP9X was significantly reduced, and the co‐localization area of Caspase‐9 and USP9X was significantly increased (Figure 6C,D); After the inhibition of USP9X expression, the cell Tunel, Annexin V and PI staining were significantly enhanced (Figure 6E,F), the apoptosis of hippocampal neurons was significantly increased, and significant late apoptotic characteristics appeared.

### 3.7 Inhibition of USP9X Expression Leads to Autophagy Disorders in Neurons

**FIGURE 7:**
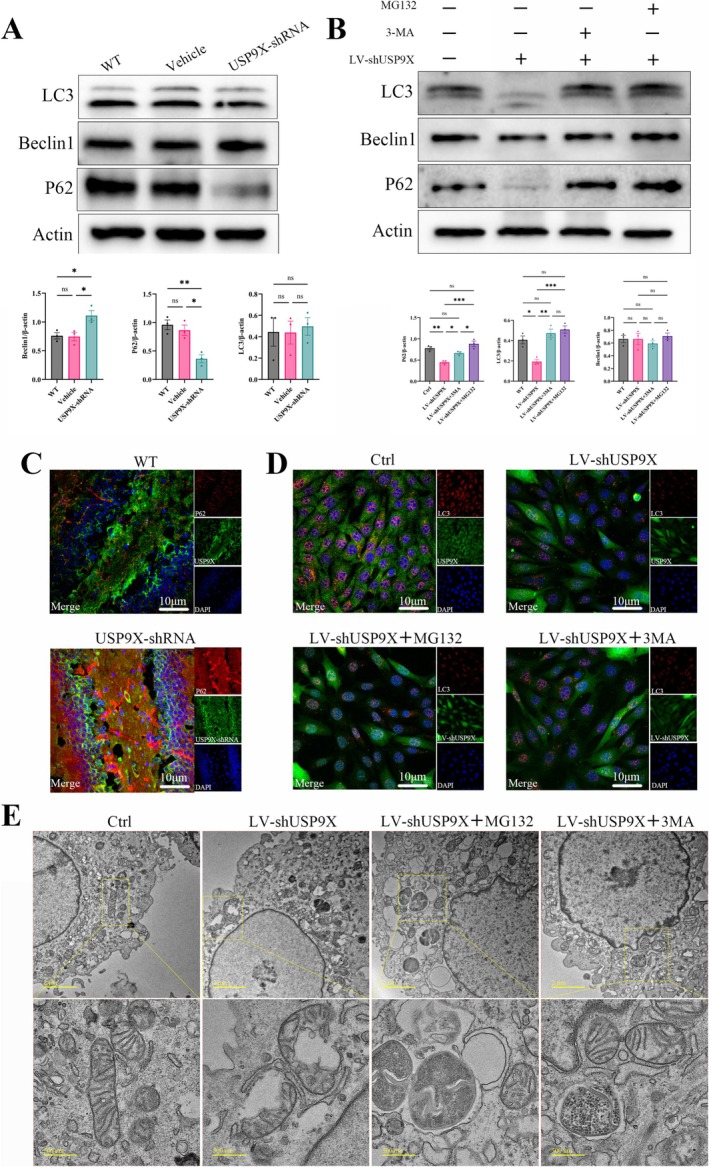
The effect of USP9X inhibition expression on neuronal autophagy. (A): western blotting results and quantification of autophagy‐related markers of tissue proteins; (B): western blotting results and quantification of HT22 cells; (C): immunofluorescence staining of hippocampal tissue; (D): immunofluorescence staining of HT22 cells; (E): results of cell scanning electron microscopy. *p < 0.05, **p < 0.01, ***p < 0.001.

P62, an autophagy substrate protein, had significantly decreased expression levels after the inhibition of USP9X expression. The expression level of Beclin‐1 was increased compared with that of WT mice, while the content of LC3 did not change significantly (in Figure 7A, for LC3/β‐actin, *F* (2, 6) = 0.08433 and *p* = 0.9202, and there is no significant difference. For Beclin1/β‐actin, *F* (2, 6) = 7.910 and *p* = 0.0208; for P62/β‐actin, *F* (2, 6) = 15.07 and *p* = 0.0046, and there are significant differences). Moreover, the expression levels of P62 and LC3B in cells were both decreased significantly (Figure 7C). To explore the mechanism by which autophagy changes after the inhibition of USP9X expression, autophagy inhibitor 3‐MA or proteasome inhibitor MG132 was added to HT‐22 cells with inhibited USP9X expression. The results showed that the expressions of P62 and LC3B both recovered (in Figure 7B, for Beclin1/β‐actin, *F* (3, 8) = 0.6286 and *p* = 0.6167, and there is no significant difference. For LC3/β‐actin, *F* (3, 8) = 16.53 and *p* = 0.0009; for P62/β‐actin, *F* (3, 8) = 17.84 and *p* = 0.0007, and there are significant differences). By analyzing the changes in mitochondrial morphology and autolysosomes using transmission scanning electron microscopy, obvious swelling and rupture was found to occur in the mitochondria of cells after inhibition of USP9X expression, but the damaged mitochondria were not cleared by autolysosomes. After the addition of MG132, the mitochondrial morphology returned to normal, obvious autophagy occurred, and the numbers of autophagosomes and autolysosomes increased (Figure 7E). After the addition of 3‐MA, the mitochondrial morphology and the number of autolysosomes also returned to the normal level. The above results indicate that P62 and LC3 are not only affected by the autolysosome pathway but also degraded through the proteasome pathway, thereby causing autophagy disorders in neurons after the downregulation of USP9X expression (Figure 7E).

## 4 Discussion

### 4.1 The Downregulation of USP9X in Hippocampal Neurons Leads to Pathological Changes Similar to AD in Mice

AD, the most common type of neurodegenerative dementia, accounts for 60%–80% of cases. It is characterized by progressive and irreversible brain degeneration, along with a decline in cognitive and memory functions. Its characteristic pathological features include β–amyloid (Aβ) deposition, neurofibrillary tangles, and synaptic loss [23, 24]. Although it has been discovered for over a century, the causes of its onset and complex molecular mechanisms remain incompletely understood. USP9X belongs to the deubiquitinating protease family within the ubiquitin‐proteasome system. Previous studies have shown that the dysregulation of this system impairs cognitive, memory, and neuronal functions in mice. Moreover, the dysregulation of USP9X expression is associated with various neurodevelopmental disorders and neurodegenerative diseases. Public transcriptome databases indicate that its expression is closely linked to the pathogenesis of AD [15, 25].

Behavioral experiments have demonstrated that after inhibiting the expression of USP9X in the hippocampus, mice exhibit impaired long‐term and short‐term memory in the eight‐arm maze test. The time taken to find the platform is significantly prolonged in the Morris water maze test, indicating impaired spatial memory ability. The number of sniffs and the time spent sniffing the novel object are reduced in the novel object recognition test, suggesting impaired cognitive function. In addition, the rotarod test for fatigue, open field test, and pole test show that the motor function remains unaffected; thus, indicating that the inhibition of USP9X expression significantly impairs the cognitive and memory functions of mice, and their behavioral manifestations are consistent with the dementia type of AD.

This study delved deeply into the associations among the impairment of cognitive and memory functions in mice caused by the down‐regulation of USP9X expression, AD‐like pathological changes, and neuronal functions. It was found that USP9X showed obvious co‐localization and protein–protein interactions with Tau and p–Tau proteins in the mouse brain. After the inhibitory expression of USP9X, the expressions of AD‐related markers Tau, p–Tau, and β–β‐amyloid precursor protein APP increased significantly. Tau protein was hyperphosphorylated, and β–β‐amyloid precursor protein was deposited. These changes were initially consistent with the AD‐like pathological features, suggesting that they might lead to neuronal death and synaptic loss.

### 4.2 The Influence of USP9X on the Functional State of Neurons

AD is characterized by prominent cognitive impairment and synaptic failure [26, 27]. Neurodegeneration is negatively correlated with synaptic plasticity and neuronal function [28]. Impairment of homeostatic regulation is associated with synaptic instability during the progression of ad [29]. Golgi staining revealed that after the inhibitory expression of USP9X, the number of mature dendritic spines of neurons in the dentate gyrus of the mouse hippocampus decreased significantly, while the number of immature dendritic spines increased, and the dendritic spine density was significantly down‐regulated. Nissl staining indicated that the number of Nissl bodies decreased significantly, the protein synthesis function of neurons was impaired, and the synaptic plasticity and synaptic homeostasis of hippocampal neurons were significantly damaged, with the physiological state and function being impaired.

Glutamate (Glu) is the major excitatory neurotransmitter in the central nervous system [30], and its homeostasis is maintained by synaptic vesicle membrane transporters. Mammals have three highly homologous vesicular glutamate transporters, namely Vglut 1–3, among which Vglut1 accounts for the largest proportion [31]. The inhibitory neurotransmitter gamma‐aminobutyric acid (GABA) is synthesized by two subtypes of glutamate decarboxylase (GAD), GAD65 and GAD67 [32]. GAD67 produces more than 90% of GABA, which is associated with various neurological diseases and plays a key role in maintaining the excitatory/inhibitory balance [33]. The GABA‐ergic system in the brain of AD patients will change [34]. This study found that after the down‐regulation of USP9X, the marker of excitatory glutamatergic interneurons, Vglut1, significantly decreased, while the marker of inhibitory GABA‐ergic interneurons, GAD67, significantly increased. The imbalance between the two led to an excitatory/inhibitory imbalance, which may be related to the cognitive impairment with AD‐like characteristics in mice. Moreover, after the inhibitory expression of USP9X, the expression level of postsynaptic density protein 95 (PSD95) increased, which may be the result of the self‐homeostatic regulation of neurons, but it failed to rescue the impairment of neuronal functions.

### 4.3 The Connection Between AD and USP9X With Neuronal Apoptosis and Autophagy

The deposition of Aβ and Tau triggers the apoptotic pathway, leading to neuronal loss in AD. This process involves both extrinsic and intrinsic pathways and activates various proteins, such as Bcl‐2 family proteins and caspases [35]. In this study, USP9X inhibition resulted in a significant downregulation of the anti‐apoptotic proteins Bcl‐2 and myeloid cell leukemia factor 1 (MCL‐1), while the pro‐apoptotic proteins, including BAX, Caspase3, and Caspase9 were upregulated. TUNEL assay, Annexin V and PI staining confirmed late‐stage apoptosis in hippocampal neurons, consistent with the neuronal apoptosis induced by AD.

Mitochondrial autophagy dysfunction is another important factor in AD pathogenesis [36]. Disruptions in multiple stages of the autophagy‐lysosome pathway contribute to the accumulation of Aβ and hyperphosphorylated Tau, leading to synaptic dysfunction and cognitive decline. In this study, USP9X downregulation led to a significant decrease in autophagy substrate protein p62 and an upregulation of Beclin‐1, a key autophagy regulator [37, 38]. After the inhibitory expression of USP9X, the autophagy substrate protein P62 in mice was significantly down‐regulated, the autophagy regulatory protein Beclin‐1 was significantly up‐regulated, and no significant change in the expression level of LC3 occurred. Verification at the cellular level showed that the expression levels of LC3 and P62 were significantly decreased. After adding the autophagy‐lysosome inhibitor 3‐MA or the proteasome inhibitor MG132, the expression levels of P62 and LC3 returned to normal. The mitochondrial autophagy induced by the inhibitory expression of USP9X may be dually regulated by the ubiquitin‐proteasome system and the autophagy‐lysosome system, and the specific mechanism remains to be further studied. Although the exact relationship between USP9X and AD still needs in‐depth exploration, the interaction between them provides a potential early molecular therapeutic target for the intervention and treatment of AD.

## 5 Conclusion

Downregulation of USP9X expression in the mouse brain leads to decreased dendritic complexity, reduced dendritic branching, and a reduction in the dendritic spine length. Additionally, it induces significant alterations in dendritic spine morphology, number, and density, severely impairing neuronal function. USP9X suppression also disrupts the balance between excitatory and inhibitory neurons, increases neuronal apoptosis, and induces autophagy dysregulation, ultimately resulting in AD‐like cognitive and memory deficits. These findings highlight the critical role of USP9X in AD pathogenesis and suggest its potential as a biomarker for early diagnosis. However, further research is needed to elucidate the precise mechanism underlying its function in the central nervous system.

## Conflicts of Interest

The authors declare that the research was conducted in the absence of any commercial or financial relationships that could be construed as a potential conflicts of interest. All authors read and approved the final version of the manuscript.

## Data Availability

The data that support the findings of this study are available from the corresponding author upon reasonable request.
